# A Viral Dynamic Model for Treatment Regimens with Direct-acting Antivirals for Chronic Hepatitis C Infection

**DOI:** 10.1371/journal.pcbi.1002339

**Published:** 2012-01-05

**Authors:** Bambang S. Adiwijaya, Tara L. Kieffer, Joshua Henshaw, Karen Eisenhauer, Holly Kimko, John J. Alam, Robert S. Kauffman, Varun Garg

**Affiliations:** 1 Vertex Pharmaceuticals Incorporated, Cambridge, Massachusetts, United States of America; 2 Johnson & Johnson Pharmaceutical Research and Development, LLC, Raritan, New Jersey, United States of America; University of California San Diego, United States of America

## Abstract

We propose an integrative, mechanistic model that integrates in vitro virology data, pharmacokinetics, and viral response to a combination regimen of a direct-acting antiviral (telaprevir, an HCV NS3-4A protease inhibitor) and peginterferon alfa-2a/ribavirin (PR) in patients with genotype 1 chronic hepatitis C (CHC). This model, which was parameterized with on-treatment data from early phase clinical studies in treatment-naïve patients, prospectively predicted sustained virologic response (SVR) rates that were comparable to observed rates in subsequent clinical trials of regimens with different treatment durations in treatment-naïve and treatment-experienced populations. The model explains the clinically-observed responses, taking into account the IC50, fitness, and prevalence prior to treatment of viral resistant variants and patient diversity in treatment responses, which result in different eradication times of each variant. The proposed model provides a framework to optimize treatment strategies and to integrate multifaceted mechanistic information and give insight into novel CHC treatments that include direct-acting antiviral agents.

## Author Summary

Hepatitis C virus chronically infects approximately 180 million people worldwide. The treatment aim for patients chronically infected with hepatitis C is viral eradication or sustained viral response (SVR). Historical standard of care for HCV treatment was peginterferon-alfa and ribavirin. Recently, approved HCV protease inhibitors, in combination with peginterferon-alfa and ribavirin, have demonstrated higher SVR rates compared to peginterferon-alfa and ribavirin alone. As members of a novel class of compounds directly targeting hepatitis C virus, HCV protease inhibitors have different mechanisms of actions and are affected by resistance and fitness of HCV variants. The significance of these different mechanisms of action, and the interplays between resistance and viral fitness to the treatment outcome has not been elucidated. Here, we developed and validated an integrative, mechanistic model of viral dynamics in response to a combination regimen including telaprevir, peginterferon-alfa, and ribavirin. The model was developed from early studies in 478 treatment-naïve patients and its SVR rate predictions were verified in 2380 patients in subsequent studies. These results provide an example of the use of mechanistic information to the development of viral dynamic model that has been useful in the design of optimal treatment regimens.

## Introduction

Chronic hepatitis C (CHC) affects approximately 180 million people worldwide and is a frequent cause of increased risk for hepatic fibrosis, cirrhosis, hepatic failure, and hepatocellular carcinoma [1], [2], [3], [4]. The treatment objective for CHC is SVR, or viral eradication, which is considered to be a virologic cure of the infection. The previous treatment for patients with genotype 1 CHC, 48 weeks of therapy with PR (PR48); results in SVR for 42%–50% of treatment-naïve patients [5], [6]. In clinical trials, a combination therapy of telaprevir and PR treatment (TPR) achieved potent antiviral activity and higher SVR rates compared to treatment with PR alone [7], [8], [9], [10], [11], [12], [13].

As a consequence of its high replication rate and its error-prone polymerase, the HCV population in a patient exists as quasispecies. At the start of treatment with direct-acting antivirals such as telaprevir, the HCV population must be considered as a mixed population, consisting predominantly of wild-type HCV (WT) and a small population of HCV variants with varying levels of resistance to telaprevir. The resistant variants generally exist at a lower frequency than WT prior to the start of treatment [14] because they are less fit (have lower replicative capacity) [15], [16], [17], [18], [19]. Variants with lower-level resistance (3 to 25-fold increase in telaprevir IC_50_ in vitro: V36A, V36M, R155K, R155T, T54A, A156S) have higher fitness than variants with higher-level resistance (25-fold increase in telaprevir IC_50_ in vitro: A156T, A156V, V36M/R155K) [18]. These variants retain sensitivity to PR treatment in vitro [20] and in patients [16], [21], [22]. WT virus was eliminated more rapidly in the presence of telaprevir than with interferon-based regimens alone in clinical trials [23], [24].

The treatment duration required to achieve SVR is based on the time to eradicate all HCV, including WT and all variants. For PR treatment, models of viral dynamics have successfully predicted SVR rates by calculating the percentage of patients whose on-treatment HCV RNA levels reach the viral eradication limit [25], [26], [27]. For TPR treatment, because of the presence of multiple variants in the quasispecies, the time when the level of each variant within a patient reaches the viral eradication limit may vary depending on the variant's fitness and resistance, and individual patient responses to treatment. The importance of these different eradication times to treatment strategies has not been elucidated.

Here, we describe a viral dynamic model of response to TPR treatment. The model incorporates the presence of viral variants of differing degrees of resistance and fitness, and the diversity in patient responses to treatment. The viral dynamic model was improved from the previously published model [18], with 2 novel features: 1) the model integrated TPR pharmacokinetics into viral dynamics, and 2) viral dynamic parameters were estimated using a population-approach method. The model was developed using in vitro and clinical data in early studies obtained from 28 patients treated with 2 weeks of telaprevir monotherapy and 478 treatment-naïve patients treated with PR and TPR regimens. Model predictions were evaluated from the outcome data of 2380 patients. Eradication of each viral variant was simulated as a discrete event occurring at variable times during treatment: when eradicated, variants were assumed to stop replicating. If eradication of all viral variants within a simulated patient was achieved, the patient was deemed to have reached SVR.

## Results

### Model development

**Figure 1:**
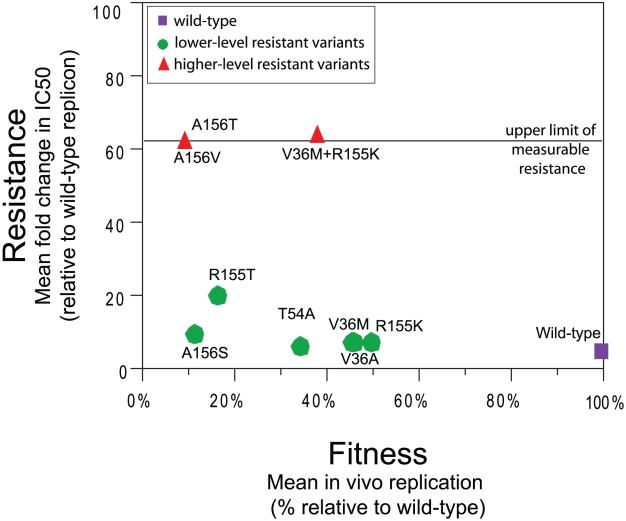
Resistance to telaprevir and fitness of variants selected during telaprevir treatment.

Model parameters were estimated from HCV RNA and drug concentration data from 478 patients who participated in early phase clinical studies (study regimens are described in Supplementary Table S1). The goodness-of-fit plot was provided in Supplementary Figure S1 and examples of fits in representative patients were provided in Supplementary Figure S2. The estimated parameters were provided in Supplementary Table S2. The estimated replicative fitness among all the variants (Figure 1) showed that the R155K variant has the highest fitness (with estimated fitness of about 50% of WT fitness), and the A156T variant has the lowest fitness (with estimated fitness of about 10% of WT fitness). Some lower-level telaprevir resistant variants (R155K, V36M, and V36A) had higher fitness than the higher-level telaprevir resistant variants (V36M/R155K, A156T). The other lower-level telaprevir resistant variants (A156S, R155T, and T54A) had lower fitness than the higher-level telaprevir resistant variants.

The individual contributions of telaprevir and PR to antiviral blockage and infected-cell clearance rates estimated from treatment-naïve population are provided in Table 1. Telaprevir contribution to production blockage ranged from −2.51−log_10_ to −2.27−log_10_ for WT and lower-level telaprevir resistant variants and −0.01−log_10_ to 0.00−log_10_ for higher-level telaprevir resistant variants, while PR treatment contributed −1.09−log_10_ for all variants. Compared to WT, lower-level telaprevir resistant variants have similar median blockages but reduced blockage in the extreme (95^th^ percentile), which occurred in patients with lower telaprevir concentrations. Infected-cell elimination rates were higher for WT and lower-level telaprevir resistant variants (0.62 d^−1^) than for higher-level telaprevir resistant variants (0.29 d^−1^). The higher elimination rates were mainly driven by higher antiviral blockage against WT and lower-level telaprevir resistant variants by telaprevir than by PR. These results suggest that the primary role of telaprevir is to block viral replication of WT and lower-level telaprevir resistant variants, and the primary role of PR is to block viral replication of higher-level telaprevir resistant variants.

**Table 1:** Contribution of telaprevir and PR treatment to the antiviral blockage and infected-cell clearance rates in treatment-naïve patient population.

Variant Name	Level of resistance	Antiviral blockage log_10_(1- ε)						infected-cell clearance *δ* (d^−1^)		
		PegIFN/RBV			telaprevir					
		Median	5^th^%	95^th^%	Median	5^th^%	95^th^%	Median	5^th^%	95^th^%
WT	None	−1.09	−1.28	−0.76	−2.27	−2.68	−1.69	0.62	0.36	1.42
R155K	Lower	−1.09	−1.28	−0.76	−2.51	−3.40	−1.28	0.65	0.36	1.54
V36A	Lower	−1.09	−1.28	−0.76	−2.39	−3.25	−1.19	0.65	0.36	1.49
A156T	Higher	−1.09	−1.28	−0.76	−0.01	−0.02	0.00	0.29	0.24	0.36
V36M/R155K	Higher	−1.09	−1.28	−0.76	0.00	−0.01	0.00	0.29	0.24	0.36

### Model evaluation

The model prediction capability was validated by comparing predicted and observed SVR rates in study regimens in which on-treatment data were used to estimate the model parameters (478 patients) and in which the model was used strictly in prediction mode (2380 patients, Supplementary Table S1). Predicted SVR rates were generated based on these inputs: (a) simulated drug concentrations and HCV RNA dynamics using parameter values re-sampled from the estimates; (b) the actual number of patients treated; (c) the number of patients who prematurely discontinued treatment; (d) the number of patients who failed to reach SVR because of other reasons (lost to follow-up, noncompliance, and withdrawal of consent); (e) the timing of treatment discontinuations; and (f) the distribution of HCV genotype (1a and 1b) for each regimen/patient population.

**Figure 2:**
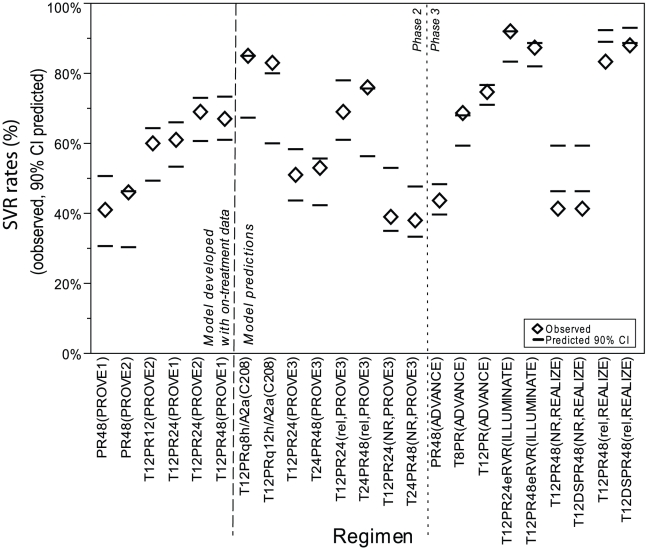
Model verification: Comparison between observed and predicted SVR rates. Notes: Population: failure, patients who were previously treated with PR48 and did not reach SVR (overall) ; rel, patients who relapsed in previous PR48 treatment; nr, patients who never had undetectable HCV RNA during previous PR48 treatment (null and partial responders). The control group (PR48) from PROVE3 was not added because by definition they were failures to PR (predicted SVR rate was zero). Some bounds are overlapping and therefore are not visible in the figure. Assumptions: The predictions of Study PROVE3 and REALIZE by prior PR responses (overall prior PR48-failure, prior PR48-relapsers, prior PR48-nonresponders) were obtained from a subset of simulated treatment-naïve patients based on HCV RNA dynamic criteria provided in the methods section.

Figure 2 shows the correspondence between observed and predicted SVR rates. In the early studies in which the on-treatment data were used to develop the model, all observed SVR rates were within the 90% confidence intervals (CIs) of the predicted rates. In subsequent studies, observed SVR rates were also consistent with predicted rates. In the subsequent Phase 2 studies, the majority of the observed SVR rates (13/14 treatment groups) were within the 90% CI bounds of the predicted rates; the other group had a rate within 3% of the nearest 90% CI bounds. In the Phase 3 treatment-naïve Studies ADVANCE and ILLUMINATE, the observed rates were within the 90% CI bounds in 4/5 groups; the other group had an observed rate that was 1% of the nearest CI bounds. In the Phase 3 treatment-experienced Study REALIZE, the observed SVR rates were all lower (by up to 7%) than the 90% CI lower bounds of the predicted rates. The discrepancy was greatest in the prior PR48-nonresponder population. In all regimens in this study, observed SVR rates were lower than predicted rates; therefore, the comparison of rates among regimens within the study is comparable between observed and predicted rates.

Despite being trained only for the treatment-naïve population, the model produced consistently predictive results even for different patient populations such as prior PR48-nonresponders and prior PR48-relapsers. The predicted SVR rates by prior PR48 responses were calculated from a subset of simulated treatment-naïve patients by classifying these patients based on their simulated HCV RNA dynamics in response to PR48 treatment, using the standard definition of PR responsiveness: prior PR48-SVR, if patients would reach SVR with PR48 treatment; prior PR48-relapser, if patients have undetectable HCV RNA at the end of PR48 treatment but not reached SVR; prior PR48-partial responder, if patients have more than 2-log_10_ HCV RNA decline at week 12 but detectable HCV RNA throughout PR48 treatment, prior PR48-null responder, if patients have less than 2-log_10_ HCV RNA decline at week 12 during PR treatment. Using the assumption that each subgroup of prior PR responses was a narrower subset of the diverse PR responsiveness of treatment-naïve population, the model was able to predict the observed higher SVR rates in prior PR48-relapser and lower SVR rates in prior PR48-nonresponders compared to rates in treatment-naïve patients.

### Viral eradication is dependent on antiviral inhibition, fitness and resistance of variant populations, and patient diversity in IFN responsiveness

**Figure 3:**
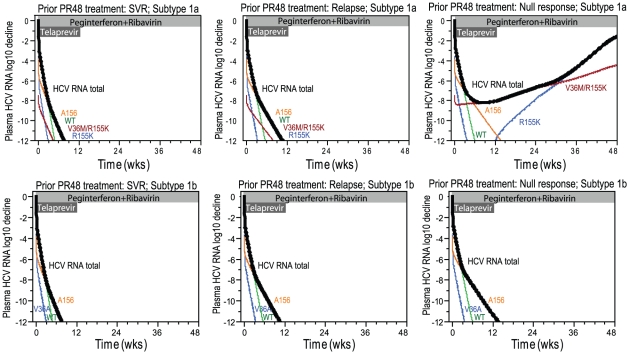
Simulated viral dynamics in patients treated with TPR, by prior PR48 responses. Notes: The simulations are for typical subtype 1a (top row) or subtype 1b (second row) patients treated with a combination regimen of 12 weeks of telaprevir and 48 weeks of peginterferon alfa-2a and ribavirin, with PR responsiveness of a typical simulated prior PR48-SVR, prior PR48-relapse, and prior PR48-null responders. The parameters were obtained from the median of parameter values in the respective category of simulated PR48 treatment. Parameters used in these simulated patients are provided in Supplementary Table S4.

To examine how viral eradication is affected by variant fitness, resistance, antiviral inhibition of each drug in the combination regimen, and patients' diversity in responses to treatment, simulations were performed for patients with 3 levels of PR responsiveness treated with 12 weeks of telaprevir in combination with 48 weeks of PR (T12PR48, Figure 3): 1) typical patient who would achieve SVR if treated with PR48 (left panel), 2) typical prior PR48-relapser (middle panel), and 3) typical prior PR48-null-responder (right panel). Simulated patients were assumed to have subtype 1a or 1b infection to provide a representative illustration. These simulations illustrate only representative examples with median responses, as there is variable PR responsiveness even within each respective group of prior PR response (the predicted SVR rates by groups of prior PR responses are provided elsewhere [28]). Patients in each HCV subtype were assumed to have the same set of major variants: for subtype 1a: WT, R155K, V36M/R155K, and A156T; for subtype 1b: WT, V36A, A156T; variants with intermediate fitness or resistance were not included (see methods). The PR responsiveness of the first 2 simulated patients with subtype 1a succeeded in eliminating all variants, but that of the last patient failed to eliminate the higher-level telaprevir resistant variant V36M/R155K. Both WT and the lower-level variant R155K were eliminated by about 6 weeks of telaprevir treatment in these 3 patients; however, the higher-level telaprevir resistant variant V36M/R155K was eliminated only in patients with better PR responsiveness (the first 2 simulated patients). In contrast, the 3 simulated patients with subtype 1b were able to reach eradication because the PR responsiveness of these patients overcame the relatively poor fitness of A156 variants (V36M/R155K variants were not present at baseline in the subtype 1b patients).

The simulation above illustrates that the variability in PR responsiveness affects the time needed to eradicate higher-level telaprevir resistant variants. For these 3 simulated patients, the time to eradicate was similar for WT and lower-level telaprevir resistant variant R155K. However, the time to eradicate higher-level telaprevir resistant variants differed by PR responsiveness: for variant A156, eradication times were 8, 11, and 13 weeks for the 3 patients; for variant V36M/R155K, the eradication time was 5 and 8 weeks for the first 2 patients, and was undefined in the last patient (because this variant was never eradicated). For the simulated null-responder patient (which as noted above, represents a median response for the null responder population), the increase in the level of V36M/R155K resulted in re-generation of R155K variant after completion of 12-week telaprevir treatment, resulting in a viral population with R155K-dominant quasispecies at week 48 (because of the higher fitness of R155K compared to V36M/R155K). However, a telaprevir duration longer than 12 weeks would also result in virologic failure but with different predominant variant in the quasispecies when failure is detected (V36M/R155K variant predominant instead of R155K variant predominant).

**Figure 4:**
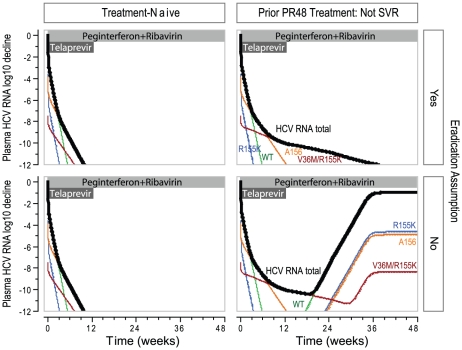
Simulated viral dynamics of typical patients on T12PR48 treatment, with and without eradication assumption. Notes: The simulations are for a typical genotype 1a patients treated with a combination regimen of 12 weeks of telaprevir and 48 weeks of peginterferon alfa-2a and ribavirin, with PR responsiveness of a typical simulated treatment-naïve and a prior PR48-non-SVR patients. The parameters for the typical PR treatment-experienced patient were obtained from median values in simulated patients who failed to reach eradication with PR48 treatment. The analyses of sensitivities to the eradication assumption were performed as follows: “Yes”, if variants cannot replicate when their levels are below the eradication limit; “No”, if variants can replicate when their levels are below the eradication limit. The limit of eradication was chosen to be 10^−5^ IU/mL, or HCV RNA decline of −12 log_10_ in a typical patient with HCV RNA baseline level of 10^7^ IU/mL.

To examine the contribution of the eradication assumption—that a variant stops replicating when its level is below the eradication limit—a simulation was performed with and without the eradication assumption. In the simulation without eradication, all variants were allowed to continue replicating even when their levels were below the eradication limit. The simulations were performed for simulated patients with 2 levels of PR responsiveness treated with T12PR: 1) typical treatment-naïve patient (Figure 4 left panels), and 2) typical patient who would not reach SVR with PR48 treatment (Figure 4 right panels). In the typical treatment-naïve patient, the predicted outcomes were the same with and without the eradication assumption: Week 48 HCV RNA levels were below the eradication limit. However, for the patient who failed to reach SVR with PR48 treatment, the outcomes differed. The dynamics in the first 12 weeks were the same: WT and lower-level telaprevir resistant variant levels reached the eradication limit by week 6. With the eradication assumption, the quasispecies left were residual higher-level telaprevir resistant variants with reduced fitness that continued to be eliminated by PR treatment, resulting in a Week 48 HCV RNA level below the eradiation limit. Without the eradication assumption, the WT HCV RNA level returned back to the baseline value around week 24 after the level reached the eradiation limit during the first 12 weeks of TPR treatment (telaprevir was only administered in the first 12 weeks). The return of HCV RNA levels after the completion of 12 weeks of telaprevir treatment with quasispecies predominately WT is rarely observed in clinical trials [8], [10], [29], supporting the eradication assumption.

**Figure 5:**
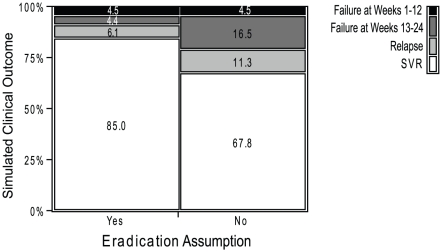
Predicted clinical outcome among treatment-naïve patients who completed T12PR24 treatment, with and without the eradication assumption. Notes: The simulations are for a simulated treatment-naïve population, with HCV genotype 1a∶1b ratio of 1∶1. The analyses of sensitivities to the eradication assumption were performed as follows: “Yes”, if variants cannot replicate when their levels are below eradication limit; “No”, if variants can replicate when their levels are below eradication limit. The simulated clinical outcomes were defined as follows: Failure at Week 1–12, HCV RNA returns back to detectable levels in the first 12 weeks (during telaprevir treatment); Failure at Week 13–24, HCV RNA levels return back to detectable level during Weeks 13–24 of therapy (during PR treatment, after completion of 12 weeks of telaprevir treatment); Relapse, HCV RNA undetectable at the end of treatment, but did not reach eradication; SVR, eradicated prior to the end of treatment. Compared to the simulated outcomes without the eradication assumption, the simulated outcomes with the eradication assumption better matched the observed clinical outcomes.

The predicted treatment outcomes with and without the eradication assumption for a population of simulated treatment-naïve patients completing a T12PR24 regimen are shown in Figure 5. Virologic outcomes were categorized as virologic failure at weeks 1–12 when TPR treatment was administered; virologic failure at Weeks 13–24 when PR treatment was administered; virologic failure after Week 24 when no treatment was administered (relapse); and SVR. Comparing simulations with and without the eradication assumption, the largest difference was observed for virologic failure between Weeks 13–24: 4.4% with eradication and 16.5% without eradication. The virologic failure rate with the eradication assumption is more consistent with rates observed in clinical trials (see discussion), supporting the eradication assumption.

## Discussion

An integrated model of viral dynamic responses to treatment with telaprevir and PR has been developed and validated by comparing predictions against observed outcomes in late-phase clinical trials. It provides a framework to integrate multi-faceted information related to this novel CHC regimen, including in vitro resistance and fitness, pharmacokinetics, viral sequencing, and viral dynamics. The framework supported decisions pertaining to treatment strategies and optimizing regimens during clinical development. The model that was based on data from early-phase trials was predictive of observed SVR rates in subsequent studies that were not used in model building.

The model also aided understanding of a novel CHC treatment regimen consisting of telaprevir and PR. It provided a consolidated picture of the interplay between the fitness and resistance of variant populations, antiviral inhibition by telaprevir and by PR treatment, and patient diversity in PR responsiveness, and connected these factors to the ultimate treatment outcome of SVR. The model suggested that the primary role of telaprevir in a TPR regimen is to eradicate WT and lower-level telaprevir resistant variants, and the complementary role of PR is to eradicate higher-level telaprevir resistant variants. Accordingly, virologic failure during the telaprevir-treatment phase has been associated predominately with higher-level telaprevir resistant variants, indicating a failure of PR to inhibit higher-level telaprevir resistant variants in some patients [9], [29]. Modeling results and analysis of viral populations derived from patients who stopped treatment prior to viral eradication [28] have led to the working hypothesis that a successful regimen should have (1) a telaprevir treatment duration sufficient to eradicate WT and most lower-level telaprevir resistant variants, and (2) a PR treatment duration sufficient to eradicate any remaining variants, particularly higher-level telaprevir resistant variants. Once WT and lower-level telaprevir resistant variants have been eradicated and higher-level telaprevir resistant variants are the dominant residual viral population, telaprevir adds no additional antiviral effect. The PR duration required to eradicate higher-level telaprevir resistant variants depends greatly on the PR responsiveness of a given patient and likely the number of residual higher-level telaprevir resistant variants. Because higher-level telaprevir resistant variants pre-exist at lower frequency than WT and have reduced fitness, a greater percentage of patients can be treated with a shorter duration of PR treatment in the TPR regimen than in the PR regimen. The personalization of PR durations for patients treated with T12PR treatment has been demonstrated in those who achieved early virologic response in clinical trials [11], [12].

Data and modeling analyses suggest different eradication times for variants with varying fitness and resistance, leading to different optimal treatment durations of telaprevir and PR treatment. Modeling analysis showed that a higher percentage of patients would be expected to have virologic failure during PR treatment after the completion of 12 weeks of telaprevir treatment if simulated without viral eradication, a phenomenon that has rarely been observed in clinical trials: the virologic failure rates after 12-week of telaprevir treatment in treatment-naïve patients were 1% for the T12PR24 arm of Study PROVE2 [8] and 4.4% in the T12PR24-48 arms of ADVANCE [28], [29]. Moreover, the shorter eradication times of sensitive variants as compared to resistant variants are also consistent with the observed more rapid elimination of WT HCV in patients dosed with telaprevir as compared to those typically observed in PR treatment [23], [24].

The model produced consistently predictive results for different prior PR48-treatment-failure populations despite being trained only for the treatment-naïve population. This finding supports the hypothesis that a treatment-naive population contains several types of patients with differing PR responsiveness, and suggests that a model estimated from the treatment-naive population can be used to predict results for populations with different PR responsiveness. In the 2 studies in the treatment-experienced population (Studies PROVE3 and REALIZE), the predicted and observed SVR rates were generally consistent: comparable SVR rates in PROVE3 and slightly higher predicted SVR rates compared to those rates observed in REALIZE. The discrepancy is greatest in the prior nonresponder population. The discrepancy in the REALIZE study may arise from a limitation of the model: that the underlying parameters constituting PR responsiveness were assumed to be continuously distributed in treatment-naïve population, while the actual parameters may be more discrete and based on other factors such as the IL28B genotypes [30], which has been reported to produce different viral dynamics in response to PR treatment [31], [32]. Alternatively, the discrepancy may be attributed to a higher proportion of patients with adverse prognostic factors for achieving SVR (e.g., advanced liver disease) enrolled in REALIZE, whereas the predictions were generated from the dataset that contained treatment-naïve patients with fewer of these adverse factors. In the modeling described here, adverse factors were not formally examined as covariates because of the limited data available from the early studies.

In summary, the proposed model served as a framework in integrating information from multiple sources and was useful in supporting decision-making for the optimization of treatment strategies during clinical development. The model provided insights to help design novel treatment regimens of combination therapy with telaprevir, peginterferon alfa-2a and ribavirin for CHC treatment, and may be useful for evaluating future CHC treatment regimens that include direct-acting antiviral agents.

## Methods

### Ethics statement

The study protocols and informed consent forms were reviewed and approved by ethics committees or institutional review boards for each clinical research site before initiation of studies at that site. Written informed consent was obtained in accordance with the Declaration of Helsinski.

### Data source

The model was developed from HCV RNA and drug concentration from a total of 478 patients treated with PR and TPR regimens in early studies of telaprevir. The model was validated using outcomes from 2380 patients in later studies. The list of studies is provided in Supplementary Table S1. The study design, enrollment criteria, and primary results have been published elsewhere [7], [8], [9], [10], [11], [12], [13], [33]. Only quantifiable HCV RNA data were used in the estimation. Additional limitations were implemented: 1) for PR regimens, only HCV RNA data up to time when the first dose modifications of either peginterferon or ribavirin were used (or end of the treatment) to evaluate the PR responses with one dose level; and 2) for TPR regimens, only patients with WT-dominant quasispecies (98% of patients) were included because few patients (2%) had resistant-variant dominant quasispecies. While the model can be applied to the patients with resistant-variant dominant quasispecies, the small number of patients in this dataset prevented us from making accurate conclusion regarding the comparability of the fitness of resistant variants in these patients to those in patients with WT-dominant quasispecies.

### Model structure and estimation

The model structure is given in Equations 1–8, and the descriptions of symbols are given in Table 2. Drug pharmacokinetics were estimated from time-concentration data in early studies. Telaprevir and peginterferon alfa-2a pharmacokinetics were described by one-compartmental models and provided in Equation 8. Ribavirin pharmacokinetics were described by a 3-compartmental model, with parameters estimated using empirical Bayesian feedback from published distributions of parameter estimates [34]. Model-predicted drug concentrations were simulated based on the dosing records and pharmacokinetic model parameters and were entered into the viral dynamic model.
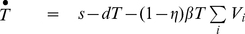
(1)

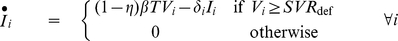
(2)

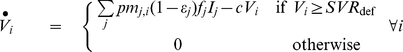
(3)

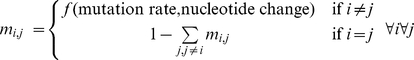
(4)

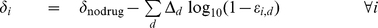
(5)

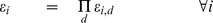
(6)

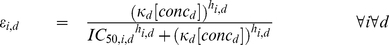
(7)

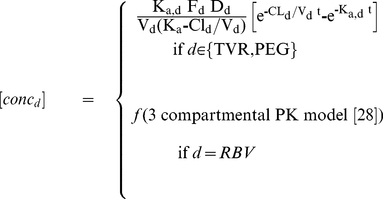
(8)


**Table 2:** Description of symbols used in Equations 1–8.

Symbol	Description
*(dot above)a variable*	time-derivative of a state variable
*T*	Healthy target cells
*s*	Target cell synthesis rate
*d*	Target cell degradation rate
*η*	Blockage of infection
*β*	Infection rate
*V*_i_ *or V*_j_	Plasma virion “*i*” or “*j*”
*I*i	Variant*-i*-infected cells
*δ*_i_	Variant*-i*-infected-cell clearance rate
Δ*_d_*	Infected-cell clearance proportionalityconstant of drug *d*
*δnodrug*	Infected-cell clearance in the absence of any drug
*p*	Production rate of wild-type (WT)
*m*_j,*i*_	Mutation rates from *Vj* to *Vi*
*ε*_i_	Blockage of production
*f*_i_	Variant-*i* fitness: production rate relative to WT
*c*	Plasma virion clearance rate
SVR_def_	HCV RNA limit of eradication
[Conc_d_]	Plasma concentration of drug *d*(*d* = telaprevir, Peg-IFN, RBV)
*Κ_d_*	Multiplier of plasma to effective concentrationsfor drug d (d = telaprevir, Peg-IFN, RBV)
*IC*_50,i,d_	IC50 of variant *i* to drug *d* (based onmeasurement in HCV replicon cells)
*h*_,i,d_	Hill coefficient of variant *i* to drug *d*(based on measurement in HCV replicon cells)
*ρ*	Ratio between *η* and *ε*_iP_
*Ka,d*	Absorption constant of drug d
*Vd*	Volume of distribution of drug d
*Cld*	Clearance of drug d
*Fd*	Bioavailability of drug d
*Dd*	Dose of drug d

**Figure 6:**
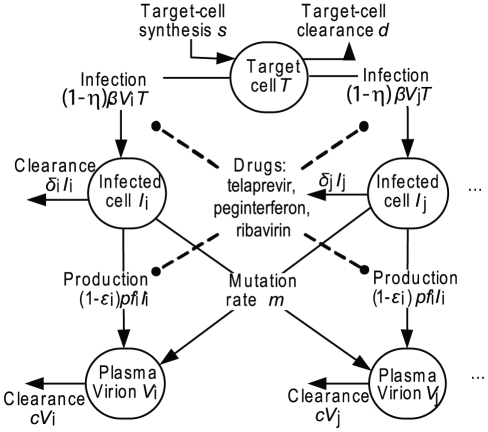
Multi-variant, viral dynamic model of a combination regimen of telaprevir and PR treatment. Parameters are defined in Supplementary Table S2 and Supplementary Table S3.

A schematic of the viral dynamic model is provided in Figure 6. Viral populations were represented as a mixture of quasispecies with varying fitness and sensitivities to telaprevir. Variant *V* represents a virion with characterized amino-acid substitution(s) in the NS3/4A protease. Variant *Vi* infects target cells (T) to form *V*-infected cells (*I*) at rate *β*T*V*. Each variant competes for the same target cells T. Target cells T also represent limited “replication space” shared by all variants; target cell T has a synthesis rate *s* and a first-order elimination *d*. In [18], a model with different representation of T (which maintain T*+I*) resulted in comparable estimates. The maximum target cells were assumed to be 10^11^ [35]. Each infected cell (*I*) produces a population of variants at production rate *pf*, with a *m*-fraction of this production mutating to produce variant *j* (*V*). The mutation rate was assumed to be 1.2 10^−4^ base^−1^ cycle^−1^ [36]. The production rate ratio (*f*) quantifies variant replicative fitness in the absence of any drug. Different production rates (*pf*), but the same infection (*β*) and clearance (*c*) rates, are assumed for different variants. This assumption is consistent with the function of the NS3/4A protease to cleave a precursor polyprotein as a crucial step in the HCV replication cycle [37]. Each drug (telaprevir, peginterferon alfa-2a, ribavirin) assumes a dual role in clearing HCV. First, each drug blocks viral production by a factor (1- *ε*). Telaprevir antiviral blockage *ε*_i,T_ is constrained to be consistent with in vitro sensitivity assay of variant *i* to telaprevir [38], [39], [40]. Blockage by peginterferon alfa-2a and ribavirin are assumed to be equal among variants, consistent with in vitro sensitivity assay. While the antiviral mechanism of ribavirin (of whether ribavirin blocked viral production or changed infectious into noninfectious viral strains) remained controversial, our data were unable to distinguish a model with a simple production blockage from a model with infectious and noninfectious viral strains [25], and therefore, a simpler model with production blockage was chosen instead of the alternative model because the alternative model would need twice as many number of variants. The blockage factors were calculated as a function of plasma concentrations of each drug (multiplied by a factor *κ* to convert plasma to effective concentrations), and the sensitivities of each variant as measured in HCV replicon cells (represented by parameters IC_50_, and hill-power values *h*). Overall blockage in the combination regimen assumed additive (in logarithmic scale) blockages of each drug. The second role of each drug is to enhance the infected-cell clearance *δ*. WT *δ*_WT_ values were up to 10-times higher in patients dosed with telaprevir than in patients treated with interferon-based regimen alone [41], [42]. These observations were represented into the model by assuming that *δ* increased proportionally with *log*_10_(1-*ε*) [18]. The enhanced *δ* may be attributed to increases in infected-cell clearance or uncovering of intracellular viral RNA [27]. Consistently, as these mechanisms may not be specific to direct-acting antivirals, the enhancement may also be observed for interferon if its effectiveness is high enough.

### Model assumptions and limitations

The HCV variants used in this model was based on the major variants detected in clinical studies: one major variant with the highest fitness for each of the resistant groups (lower-level and higher-level resistance) and the nucleotide changes from WT. Subtypes 1a and 1b were modeled separately because when telaprevir was administered in monotherapy, different sets of resistant variants emerged [15], [16]. All patients with the same subtype were assumed to have the same set of major variants: for subtype 1a: WT, R155K, V36M/R155K, and A156T/V; for subtype 1b: WT, V36A, A156T/V. The frequency of these variants prior to treatment was calculated by assuming a steady-state condition. The intermediate-resistant variants R155T/I and other minority variants observed in a few patients were not included in the model used to generate predictions because of lack of data to estimate their fitness. Including these variants in the model was expected to result in only small changes in the SVR rates, because these variants appeared to be less fit than the variants used in the model [18]. The parameters related to the antiviral activity of peginterferon alfa and ribavirin were correlated because the current training dataset contained data from regimens where peginterferon and ribavirin were administered simultaneously. Because of the data limitation, the proportionality constant related to the enhanced infected-cell clearance for ribavirin is assumed to be equal to the constant for peginterferon.

### Calculation of predicted SVR rates

SVR rates were predicted by evaluating simulated HCV RNA dynamics and entering the observed patient disposition into the model. The predicted HCV RNA dynamics for treatment-naïve patients were generated by simulations, with parameters re-sampled from the distributions of estimates in Supplementary Table S2, truncated by lower and upper bounds (bounds were obtained from the extreme values of the observed individual estimates). Dosing compliance was assumed to be 100%. Ribavirin dose modification followed the observed modification in the training dataset. A simulated patient was considered to achieve eradication (or SVR) if the overall HCV RNA level by the end of treatment was below 1 copy in the body [25] (or reached a 12-log decline from baseline in HCV RNA, assuming a baseline value of 10^7^ IU/mL).

Predicted SVR rates for different categories of PR responsiveness (SVR with PR48, prior PR48-non-SVR, prior PR48-relapser, prior PR48-nonresponder, prior PR48-null responder) were generated by simulating HCV RNA dynamics to PR48 treatment, and by filtering the responses with the respective PR responsiveness criteria. The categories of PR responsiveness followed these criteria: SVR with PR48, if patient's viral load reached eradication by the end treatment; prior PR48-non-SVR, if patient's viral load did not reach eradication by the end of treatment; prior PR48-relapser, if patient's viral load was undetectable by the end of treatment but did not reach eradication; prior PR48-nonresponder, if patient's viral load was always detectable during treatment; prior PR48-null-responder, if patient's viral load at week 12 declined <2−log_10_.

### Numerical implementation

Drug concentrations were estimated or simulated using a Bayesian approach implemented in NONMEM version 6. Viral dynamic model was implemented in Jacobian® software version 4.0 (RES group, Inc., Cambridge, MA).

## Supporting Information

Figure S1 **Goodness of fit plot of HCV RNA Log10 decline.** DV = observed values; IPRED = model-fit values; IWRES = residual values.
(DOC)

Figure S2 **Example of representative fits.** Plasma concentration of telaprevir and ribavirin is expressed in µg/mL; serum concentration of Peg-IFN is expressed in ng/mL. Two-step parameter estimations were performed for each patient: 1) estimation of PK parameters; and 2) estimation of HCV RNA dynamic parameters with PK parameters as inputs. For estimation of PK parameters, the following parameters were estimated from PK measurements of telaprevir and of Peg-IFN: Ka, Cl, and V; and these parameters were estimated from PK measurements of RBV: Ka, Cl, Q3, Q4, V2, V3, V4 [34]. For patients treated with PR regimen, the following parameters were estimated from HCVRNA measurements: c, δ_P_, κ_P_, κ_R_, and ρ. For patients treated with TPR g1b regimen, the following parameters were estimated from HCV RNA measurements: c, δ_T,_,δ_P_, κ_T_, κ_P_, κ_R_, ρ, f_R155K_, f_A156T_, f_V36MR155K_. For patients treated with TPR g1b regimen, the following parameters were estimated from HCV RNA measurements: c, δ_T,_,δ_P_, κ_T_, κ_P_, κ_R_, ρ, f_V36A_, f_A156T_.
(EPS)

Table S1 **Source and description of study regimens used for model estimation and verification.**
(DOC)

Table S2 **Final parameter estimates of pharmacokinetics and viral dynamics from data obtained in 28 patients treated with 2 weeks of telaprevir in monotherapy and in 478 treatment-naïve patients treated with PR and TPR regimens.** Each parameter assumed lognormal distribution, of which log_10_ of mean and variance were provided.
(DOC)

Table S3 **Parameters obtained from literature or assumed.** Assumed values have been verified not to change the conclusions of the results.
(DOC)

Table S4 **Parameter values used in** **Figure 3** **.**
(DOC)
