# Supplementary material for: A Viral Dynamic Model for Treatment Regimens with Direct-acting Antivirals for Chronic Hepatitis C Infection
Source: PLoS Comput Biol. 2012 Jan 5;8(1):e1002339. doi: 10.1371/journal.pcbi.1002339 (PMC3252270; doi:10.1371/journal.pcbi.1002339)
Supplement: Figure S1 — Goodness of fit plot of HCV RNA Log10 decline. DV = observed values; IPRED = model-fit values; IWRES = residual values. (DOC) [file pcbi.1002339.s001.doc]

Supplementary Figure S1 Goodness of Fit Plot of HCV RNA Log10 Decline

DV= observed values; IPRED=model-fit values; IWRES= residual values

| (a) for patients on Peg-IFN and RBV treatment |
| --- |
|  |
| (b) for subtype 1a patients on telaprevir, Peg-IFN, and RBV treatment |
|  |
| (c) for subtype 1b patients on telaprevir, Peg-IFN, and RBV treatment |
|  |
