# Supplementary material for: A Viral Dynamic Model for Treatment Regimens with Direct-acting Antivirals for Chronic Hepatitis C Infection
Source: PLoS Comput Biol. 2012 Jan 5;8(1):e1002339. doi: 10.1371/journal.pcbi.1002339 (PMC3252270; doi:10.1371/journal.pcbi.1002339)
Supplement: Table S1 — Source and description of study regimens used for model estimation and verification. (DOC) [file pcbi.1002339.s003.doc]

Supplementary Table S1 Source and Description of Study Regimens Used for Model Estimation and Verification

| Study Name | Phase | Population | Regimens | N | Note |
| --- | --- | --- | --- | --- | --- |
| 101[33] | 1 | Treatment-naïve and prior PR48-treatment failure | Telaprevir monotherapy (14 days)  450mg every 8h  750mg every 8h  1250mg every 12h | 28 | **Estimation**: on-treatment data;  **Prediction**: observed SVR rates were compared against predicted SVR rates |
| PROVE1[7] | 2 | Treatment-naïve | PR48  T12PR24  T12PR48 | 75  79  79 |
| PROVE2 [8] | 2 | Treatment-naïve | PR48  T12PR12  T12PR24 | 82  82  81 |
| C208[10] | 2 | Treatment-naïve | T12PR24-48 (telaprevir: 750mg every 8 h)  T12PR24-48 (telaprevir: 1125mg every 12 h) | 40  40 | **Prediction**: Observed SVR rates were compared against predicted SVR rates |
| PROVE3[9] | 2 | Prior PR48-treatment failure  *Prior PR48-nonresponder*  *Prior PR48-relapser* | T12PR24  T24PR48 | 115  113 |
| ADVANCE[11] | 3 | Treatment-naïve | PR48  T8PR24-48  T12PR24-48 | 361  364  363 |
| ILLUMINATE[12] | 3 | Treatment-naïve | T12PR24  T12PR48 | 162  160 |
| REALIZE[13] | 3 | Prior PR48-treatment failure  *Prior PR48-nonresponder*  *Prior PR48-relapser* | PR48  T12PR48  T12DSPR48 | 132  266  264 |

Abbreviations: P: peginterferon alfa-2a; PR_24-48_, PR duration based on HCV RNA undetectability at Weeks 4 and 12; R: ribavirin; T: telaprevir; T12DS: 4-week delayed start of telaprevir treatment;

PR48: 48 weeks of PR treatment

T12PR24: 12 weeks of TPR treatment + 12 weeks of PR treatment

T12PR12: 12 weeks of TPR treatment

T12PR_24-48_: 12 weeks of TPR treatment + 12 or 36 weeks of PR treatment (depending on undetectability at Weeks 4 and 12 or eRVR)

T12DSPR48: 12 weeks of telaprevir and 48 weeks of PR treatment; 4-week delayed start of telaprevir treatment
