# Supplementary material for: A Viral Dynamic Model for Treatment Regimens with Direct-acting Antivirals for Chronic Hepatitis C Infection
Source: PLoS Comput Biol. 2012 Jan 5;8(1):e1002339. doi: 10.1371/journal.pcbi.1002339 (PMC3252270; doi:10.1371/journal.pcbi.1002339)
Supplement: Table S2 — Final parameter estimates of pharmacokinetics and viral dynamics from data obtained in 28 patients treated with 2 weeks of telaprevir in monotherapy and in 478 treatment-naïve patients treated with PR and TPR regimens. Each parameter assumed lognormal distribution, of which log10 of mean and variance were provided. (DOC) [file pcbi.1002339.s004.doc]

Supplementary Table S2 Final Parameter Estimates of Pharmacokinetics and Viral Dynamics from Data Obtained in 28 Patients Treated with 2 weeks of Telaprevir in Monotherapy and in 478 Treatment-Naïve Patients Treated with PR and TPR Regimens

Each parameter assumed lognormal distribution, of which log_10_ of mean and variance were provided.

| **Parameter Name** | **Unit** | | **Log**B**_10_**_B_ **of Mean** | | **IIV (variance)** | | **Lower Bound**TPF**[[1]](#footnote-2)**FPT | | **Upper Bound**aTPaFP | | **Data Source** |
| --- | --- | --- | --- | --- | --- | --- | --- | --- | --- | --- | --- |
| **Parameters of viral dynamic model** | | | | | | | | | | | |
| Plasma virion clearance *c* | h^-1^ | | -1.78E-01 | | 6.25E-02 | | -1.01E+00 | | 7.47E-01 | | [7,8] |
| Enhancement factor of infected-cell clearance by telaprevir *δ*_T_ | h^-1^ | | -4.21E-01 | | 6.50E-01 | | -4.12E+00 | | 2.16E+00 | | [7,8] |
| Enhancement factor of infected clearance by PegIFN *δ*_P_ | h^-1^ | | -4.63E-01 | | 5.82E-01 | | -4.25E+00 | | 2.29E+00 | | [7,8] |
| Multiplier of plasma to effective concentrations for telaprevir *κ*_T_ | unitless | | 1.85E-01 | | 1.68E-01 | | -1.88E+00 | | 1.45E+00 | | [7,8] |
| Multiplier of plasma to effective concentrations for peginterferon alfa-2a *κ*_P_ | unitless | | -2.19E-01 | | 4.76E+00 | | -1.34E+01 | | 5.45E+00 | | [7,8] |
| Multiplier of plasma to effective concentrations for ribavirin *κ*_R_ | unitless | | 2.63E-02 | | 7.07E-02 | | -6.61E-01 | | 2.49E+00 | | [7,8] |
| Ratio of infection blockage to production blockage ρ | unitless | | 1.08E-01 | | 2.31E-01 | | -1.41E+00 | | 3.00E+00 | | [7,8] |
| Variant R155K fitness relative to WT (subtype 1a only) | unitless | | -2.27E-03 | | 6.00E-03 | | -3.41E-01 | | 5.03E-01 | | [7,8] |
| Variant A156T fitness relative to WT | unitless | | 2.35E-02 | | 4.48E-03 | | -1.27E-01 | | 5.29E-01 | | [7,8] |
| Variant V36M/R155K fitness relative to WT (subtype 1a only) | unitless | | 5.02E-03 | | 5.73E-03 | | -6.32E-01 | | 1.88E-01 | | [7,8] |
| Variant V36A fitness relative to WT (subtype 1b only) | unitless | | 9.82E-03 | | 2.51E-03 | | -1.27E-02 | | 2.98E-01 | | [7,8] |
| **Parameters of pharmacokinetic models** | | | | | | | | | | | |
| Absorption of PegIFN Ka_P_ | h^-1^ | | -6.08E+00 | | 4.23E-01 | | -9.08E+00 | | -3.94E+00 | | [7,8,9] |
| Clearance of PegIFN Cl_P_ | L h^-1^ | | -2.77E+00 | | 8.17E-01 | | -4.45E+00 | | 1.38E+00 | | [7,8,9] |
| Volume of peginterferon alfa-2a V_P_ | L | | 3.13E-01 | | 3.42E-01 | | -2.50E+00 | | 3.68E+00 | | [7,8,9] |
| Absorption of ribavirin Ka_R_ | | h^-1^ | | -2.66E-01 | | 3.40E-04 | | -3.37E-01 | | -1.37E-01 | [34] |
| Clearance (central compartment) of ribavirin Cl_R_ | | L h^-1^ | | 2.88E+00 | | 1.64E-02 | | 2.42E+00 | | 3.22E+00 | [34] |
| Volume (central compartment) of ribavirin V2_R_ | | L | | 6.16E+00 | | 4.22E-03 | | 5.82E+00 | | 6.36E+00 | [34] |
| Intercompartmental clearance (compartment 3) of ribavirin Q3_R_ | | h^-1^ | | 3.54E+00 | | 4.65E-05 | | 3.51E+00 | | 3.58E+00 | [34] |
| Volume (compartment 3) of ribavirin V3_R_ | | L | | 8.38E+00 | | 2.68E-02 | | 7.82E+00 | | 8.75E+00 | [34] |
| Intercompartmental clearance (compartment 4) of ribavirin Q4_R_ | | h^-1^ | | 4.58E+00 | | 3.14E-04 | | 4.45E+00 | | 4.63E+00 | [34] |
| Volume (compartment 4) of ribavirin V4_R_ | | L | | 6.77E+00 | | 2.74E-05 | | 6.73E+00 | | 6.79E+00 | [34] |
| Bioavailability of central compartment of ribavirin F1_R_ | | unitless | | 2.85E-02 | | 2.33E-02 | | -3.80E-01 | | 6.67E-01 | [34] |
| Absorption of telaprevir Ka_T_ | | h^-1^ | | -7.03E-01 | | 8.64E-03 | | -7.82E-01 | | -5.94E-01 | [7,8] |
| Clearance of telaprevir Cl_T_ | | h^-1^ | | 3.50E+00 | | 6.32E-02 | | 2.69E+00 | | 4.59E+00 | [7,8] |
| Volume of telaprevir V_T_ | | L | | 5.93E+00 | | 1.09E-01 | | 4.99E+00 | | 7.20E+00 | [7,8] |
| Bioavailability of central compartment of telaprevir F1_T_ | | unitless | | 1.00E+00 | | 0.00E+00 | | na | | na | [7,8] |

1. TP?PT Bounds were used to truncate distribution [↑](#footnote-ref-2)
