# Supplementary material for: A Viral Dynamic Model for Treatment Regimens with Direct-acting Antivirals for Chronic Hepatitis C Infection
Source: PLoS Comput Biol. 2012 Jan 5;8(1):e1002339. doi: 10.1371/journal.pcbi.1002339 (PMC3252270; doi:10.1371/journal.pcbi.1002339)
Supplement: Table S3 — Parameters obtained from literature or assumed. Assumed values have been verified not to change the conclusions of the results. (DOC) [file pcbi.1002339.s005.doc]

Supplementary Table S3 Parameters Obtained from Literature or Assumed

Assumed values have been verified not to change the conclusions of the results.

| **Parameter Name** | **Unit** | **Value** | **Source** |
| --- | --- | --- | --- |
| Mutation rate *m* | Base^-1^ cycle^-1^ | 1.20E-04 | [36] |
| Baseline infected-cell clearance rates *δ*B_nodrugB_ | h^-1^ | 5.20E-03 | [41] |
| Target-cell synthesis *s* | cells h^-1^ | 8.40E+08 | [18,33]^b^ |
| Reproductive ratio *R*B_0,WTB_ | unitless | 5.52E+01 | [18,33]^b^ |
| Infection rate *β* | h^-1^ virion^-1^ | 5.00E-02 | [18,33][[1]](#footnote-2) |
| Maximum replication space *T*_max_ (Note: target cell clearance rate *d* is calculated from the equation *T*_max_=*s/d*) | cells | 1.00E+11 | [35]^b^ |
| IC_50_ of telaprevir to WT IC_50, WT,T_ | uM | 1.06E+00 | [38,39,40] |
| Hill factor of telaprevir to WT h_WT,T_ | unitless | 1.66E+00 | [38,39,40] |
| IC_50_ of telaprevir to V36A IC_50,R155K,T_ | uM | 4.99E+00 | [39,40] |
| Hill factor of telaprevir to V36A *h*_R155K,T_ | unitless | 3.46E+00 | [39,40] |
| IC_50_ of telaprevir to R155K IC_50,R155K,T_ | uM | 4.76E+00 | [39,40] |
| Hill factor of telaprevir to R155K *h*_R155K,T_ | unitless | 3.54E+00 | [39,40] |
| IC50 of telaprevir to A156T IC_50,A156T,T_ | uM | 1.00E+03 | [39,40] |
| Hill factor of telaprevir to A156T *h*_A156T,T_ | unitless | 1.00E+00 | [39,40] |
| IC50 of telaprevir to V36M/R155K IC50_V36M/R155K,T_ | uM | 1.43E+02 | [39,40] |
| Hill factor of telaprevir to V36M/R155K *higher-level telaprevir resistant variant*_36M/R155K,T_ | unitless | 3.54E+00 | [39,40] |
| IC50 of peginterferon alfa-2a IC50_P_ | uM | 2.51E+02 | [39,40] |
| Hill factor of peginterferon alfa-2a h_P_ | unitless | 4.41E-01 | [39,40] |
| IC50 of ribavirin IC50_R_ | uM | 1.00E+00 | Assumed value |
| Hill factor of ribavirin h_R_ | unitless | 1.00E+00 | Assumed value |

1. These parameters were estimated from HCV RNA dynamics in patients dosed with 2 weeks of telaprevir in monotherapy. The data source was provided in Ref 1. Reesink HW, Zeuzem S, Weegink CJ, Forestier N, van Vliet A, et al. (2006) Rapid Decline of Viral RNA in Hepatitis C Patients Treated With VX-950: A Phase Ib, Placebo-Controlled, Randomized Study. Gastroenterology 131: 997-1002. and the structure of the model was published in Ref 2. Adiwijaya BS, Herrmann E, Hare B, Kieffer T, Lin C, et al. (2010) A Multi-Variant, Viral Dynamic Model of Genotype 1 HCV to Assess the In Vivo Evolution of Protease-Inhibitor Resistant Variants. People Library of Science Computational Biology 6: e1000745. Because of the lack of direct measurement of target cells nor productively infected cells, we were able to estimate only the overall viral replication rates, or the basic reproductive ratio *R**_0_*_,WT_= *pβT*_max_/*(cδ)*. If measurements of infected cells and target cells were available, parameters *p*, *β, s,* and *d* may be adjusted to match the measured infected and target cells while maintaining the constraint that *R*_0,WT_ remains constant. For a given R_0,WT_ value, some degree of freedoms exist in choosing *p*, *β, s,* and *d* , while clearance parameters *c* and *δ* would be constrained by the decline kinetics. Numerically, this is implemented by fixing parameters *β* and the *s/d* ratio to the values provided in Supplementary Table S3, normalized HCV RNA levels to the baseline value, and scaled *T, T*_max_*,* *I* and *s* values by a factor of 10^10^. When measurements of target and infected cells become available, different values of *s* and *T*_max_ may adjusted as appropriate to maintain R_0,WT_ values and the changes would result in the same HCV RNA dynamics. Please refer to 2. Adiwijaya BS, Herrmann E, Hare B, Kieffer T, Lin C, et al. (2010) A Multi-Variant, Viral Dynamic Model of Genotype 1 HCV to Assess the In Vivo Evolution of Protease-Inhibitor Resistant Variants. People Library of Science Computational Biology 6: e1000745. for the details of this estimation method. [↑](#footnote-ref-2)
