# Supplementary material for: A Viral Dynamic Model for Treatment Regimens with Direct-acting Antivirals for Chronic Hepatitis C Infection
Source: PLoS Comput Biol. 2012 Jan 5;8(1):e1002339. doi: 10.1371/journal.pcbi.1002339 (PMC3252270; doi:10.1371/journal.pcbi.1002339)
Supplement: Table S4 — Parameter values used in Figure 3 . (DOC) [file pcbi.1002339.s006.doc]

Supplementary Table S4 Parameter Values Used in Figure 3

| **Parameter Name** | **Unit** | **Log**B**_10_**_B_ **of Mean** | **Subtype 1a** | | | **Subtype 1b** | | |
| --- | --- | --- | --- | --- | --- | --- | --- | --- |
|  |  |  | **Prior SVR** | **Prior Relapser** | **Prior Null** | **Prior SVR** | **Prior Relapser** | **Prior Null** |
| Plasma virion clearance *c* | h^-1^ | -1.78E-01 | -0.176 | -0.182 | -0.174 | -0.176 | -0.182 | -0.174 |
| Enhancement factor of infected-cell clearance by telaprevir *δ*_T_ | h^-1^ | -4.21E-01 | -0.423 | | | | | |
| Enhancement factor of infected clearance by PegIFN *δ*_P_ | h^-1^ | -4.63E-01 | -0.254 | -0.671 | -0.655 | -0.254 | -0.671 | -0.655 |
| Multiplier of plasma to effective concentrations for telaprevir *κ*_T_ | unitless | 1.85E-01 | 0.179 | | | | | |
| Multiplier of plasma to effective concentrations for peginterferon alfa-2a *κ*_P_ | unitless | -2.19E-01 | 1.265 | -0.173 | -2.180 | 1.265 | -0.173 | -2.180 |
| Multiplier of plasma to effective concentrations for ribavirin *κ*_R_ | unitless | 2.63E-02 | 0.044 | 0.034 | -0.025 | 0.044 | 0.034 | -0.025 |
| Ratio of infection blockage to production blockage \rho | unitless | 1.08E-01 | -0.001 | 0.064 | 0.251 | -0.001 | 0.064 | 0.251 |
| Variant R155K fitness relative to WT (subtype 1a only) | unitless | -2.27E-03 | -0.003 | | | NA | | |
| Variant A156T fitness relative to WT | unitless | 2.35E-02 | 0.023 | | | 0 | | |
| Variant V36M/R155K fitness relative to WT (subtype 1a only) | unitless | 5.02E-03 | 0.005 | | | NA | | |
| Variant V36A fitness relative to WT (subtype 1b only) | unitless | 9.82E-03 | NA | | | 0.022 | | |
